# [Retracted] EVI‑1 acts as an oncogene and positively regulates calreticulin in breast cancer

**DOI:** 10.3892/mmr.2023.13112

**Published:** 2023-10-09

**Authors:** Lei Wu, Tianyi Wang, Dongning He, Xiaoxi Li, Youhong Jiang

Mol Med Rep 19: 1645–1653, 2019; DOI: 10.3892/mmr.2018.9796

Following the publication of this paper, it was drawn to the Editor's attention by a concerned reader that, for the Transwell cell migration and invasion assay experiments shown in Fig. 3 on p. 1650, there were several panels showing overlapping sections of data; moreover, certain of the data shown in this Figure were also strikingly similar to data appearing in different form in Fig. 4 in another article written by different authors at a different research institute [Liu J and Duan X: PA-MSHA induces apoptosis and suppresses metastasis by tumor associated macrophages in bladder cancer cells. Cancer Cell Int 17: 76, 2017].

Owing to the fact that the contentious data in the above article had already been published prior to its submission to *Molecular Medicine Reports*, the Editor has decided that this paper should be retracted from the Journal. The authors were asked for an explanation to account for these concerns, but the Editorial Office did not receive a reply. The Editor apologizes to the readership for any inconvenience caused.

